# Selenomethionine and Allicin Synergistically Mitigate Intestinal Oxidative Injury by Activating the Nrf2 Pathway

**DOI:** 10.3390/toxics12100719

**Published:** 2024-09-30

**Authors:** Yongshi Liu, Xi Lv, Heling Yuan, Xiaoming Wang, Jinhu Huang, Liping Wang

**Affiliations:** College of Veterinary Medicine, Nanjing Agricultural University, Nanjing 210095, China; lysliuyongshi@163.com (Y.L.); lvxi96@163.com (X.L.); 18336060391@163.com (H.Y.); wangxm@njau.edu.cn (X.W.); jhuang@njau.edu.cn (J.H.)

**Keywords:** SeMet, allicin, antioxidative effect, Nrf2, endoplasmic reticulum stress, intestinal barrier injury

## Abstract

Oxidative stress frequently contributes to intestinal barrier injury in animals and humans. It was reported that both Selenomethionine (SeMet) and allicin exhibit protective effects against a range of diseases caused by oxidative stress. This study aimed to investigate the synergistic antioxidant effects and underlying mechanisms of SeMet and allicin on a H_2_O_2_-induced intestinal barrier injury model using IPEC-J2 cells and mice. The results showed that H_2_O_2_ induced severe oxidative stress, including a decrease in cell viability, antioxidant level, migration capacity, and cell integrity. SeMet and allicin exhibited significant synergistic anti-oxidative effects on intestinal epithelial cells. The combined use of SeMet and allicin increased SOD activity, GSH content, and GSH/GSSG ratio while decreasing MDA, NO, and ROS content levels. Furthermore, we found that SeMet and allicin synergistically activated the nuclear factor erythroid-related factor 2 (Nrf2)-NAD(P)H dehydrogenase [quinone] 1 (NQO1) signaling pathway and down-regulated endoplasmic reticulum stress (ER stress)-related proteins. However, the synergistic antioxidative and intestinal barrier protective effects of SeMet and allicin were abolished by Nrf2 inhibitor ML385 in vitro and in vivo. In conclusion, SeMet and allicin synergistically attenuate intestinal barrier injury induced by excessively oxidative stress through the activation of the Nrf2 signaling pathway and inhibition ER stress. These findings support that the combined use of SeMet and allicin could enhance antioxidative properties and alleviate intestinal injury in further clinical practice.

## 1. Introduction

Oxidative stress occurs when reactive oxygen species (ROS) production surpasses the ROS elimination by antioxidant enzymes of cells, resulting in numerous animal and human diseases. The intestine is a vital organ for digestion and absorption and the largest immune organ in human and animals [1]. In general, the gut barrier is recognized as the first line of defense for humans and animals against invading oxidative stress irritants and other foreign substances. The selective permeability of barrier function is primarily maintained by the paracellular permeability of intestinal epithelial cells, which is mediated by tight junctions (TJs) [2], and tight junctions proteins are key proteins in repairing intestinal epithelial cells [3,4]. Intestinal epithelial cells are susceptible to exogenous ROS due to the specific gut characteristics [5].

Endoplasmic reticulum stress (ER stress) refers to the imbalance of ER lumen homeostasis because of the disorder of Ca^2+^ ions in the ER and the misfolded protein [6]. Under stress conditions, cells initiate unfolded protein response (UPR) for compensation and regulation mechanisms. UPR transmits the signal to the nucleus and cytoplasm to recover or to apoptosis based on the oxidative stress degree [7]. Furthermore, cells respond to ER stress through three classical signaling proteins in eukaryotes: inositol-requiring enzyme (IRE), protein kinase R-like ER kinase (PERK), and activating transcription factor 6 (ATF6). Commonly, the three signaling proteins are combined with glucose-regulated protein 78 (GRP78) [8]. Upon the misfolded proteins accumulated in ER, they competitively bind to GRP78 with IRE, PERK, and ATF6. The three signaling proteins dissociated from GRP78 and activated downstream signaling pathways to maintain cell homeostasis. The PERK-activated transcription factor-4 (ATF4), nuclear factor erythroid-related factor 2 (Nrf2), and C/EBP-homologous protein (CHOP) expression [9,10,11]. The Nrf2 signaling pathway, a basic leucine zipper transcription factor, synthesizes the redox reaction system to maintain cell redox balance and metabolism. The Nrf2 ECH homeodomain (Neh) 2 area on Nrf2 is combined with Kelch-like ECH-associated protein 1 (Keap1), a 69 kDa protein, in the cytoplasm; Keap1 rapidly degrades Nrf2 through the ubiquitin–proteasome system (Cullin3). ROS covalently modify Keap1 cysteine residues when cells suffer oxidative stress to change Keap1 conformation [12]. Nrf2 released from Keap1 and translocated into the nucleus to form a heterodimer [13], which activated antioxidant response element (ARE)-dependent downstream antioxidant gene expressions, such as NQO1 and superoxide dismutase (SOD) [14,15]. In order to relieve intestinal oxidative stress, protect the intestinal barrier, and enhance intestinal function, it is important to explore effective antioxidant drugs or drug combinations.

Selenium (Se) is a necessary nutrient for humans and animals in antioxidant defense, anti-inflammation, anti-tumorigenesis, immune defense, muscle metabolism, detoxification, and antiviral defense processes. Selenoproteins and Se-containing enzymes are important in redox homeostasis [16]. Selenomethionine (SeMet), a safe organic Se source, is more bioavailable than sodium selenite or Se yeast and can offer a solution to the Se puzzle in scientific Se supplements [17]. SeMet is a selenium-containing amino acid, for which the sulfur in methionine is replaced by selenium. SeMet is widely used in foods, beverages, nutraceuticals, the treatment of gastrointestinal diseases, diabetes mellitus, and Alzheimer’s disease due to its unique biological effects such as the high efficiency of antioxidants, low toxicity, and high bioavailability, which are incomparable with other organic selenium compounds. Garlic contains many sulfur compounds, making it a valuable component in both culinary and medicinal practices; allicin is the most bioactive compound in garlic. The damaged garlic tissue (caused by chewing or mashing) releases the alliinase found in the bulb into the cytoplasm under the action of cysteinase and then catalyzes the S-allyl cysteine sulfoxide into allicin [18,19,20]. Allicin has many biological and pharmacological activities (including antioxidant and anti-inflammatory activities) and can interact with intracellular thiol compounds [21]. Moreover, allicin can alleviate cyclophosphamide-caused hepatic injury by activating the Nrf2 pathway [22]. 

Se and sulfur are widely present in garlic. Also, Se belongs to the same group as sulfur in the periodic table and is therefore chemically similar [23]. Accordingly, collaborative use seems to exert a possible synergistic antioxidant effect [21]. Additionally, allicin mitigated acrylamide-induced NLRP3 inflammasome activation by inhibiting oxidative stress and ER stress in the liver of Sprague Dawley (SD) rats and in Kupffer cells [24]. Similarly, Se supplementation effectively inhibits ferroptosis and cell death induced by ER stress [25]. Meanwhile, Se alleviates cadmium-induced hepatotoxicity and relieves ER stress in piglets [26,27]. These results suggest that Se and allicin can regulate oxidative stress and attenuate ER stress through their complex regulatory networks. However, Se has a narrow therapeutic window and the toxicity margins are very delicate, and the excessive intake of SeMet often leads to toxicity [28]. Allicin is an irritant, activate transient receptor potential A1 (TRPA1), causing acute pain and other side effects [29]. Thus, the therapeutic doses of allicin need to be limited. In this case, the combination of drugs may be an effective strategy. Combined administration can not only increase the antioxidant protection effect and reduce side effects, but also reduce the administration cost. However, it remains unclear whether combining SeMet and allicin provides better protection against intestinal barrier damage by inhibiting oxidative stress and their specific protective mechanism. To elucidate the synergistic effect of SeMet and allicin, the combination index (CI) was utilized. The CI indicates the antagonism effect (CI > 1), additive effect (CI = 1), and synergism effect (CI < 1) [30]. Therefore, this study aimed to investigate the synergistic, antagonistic, and additive interactions of SeMet and allicin co-treatment using the Chou–Talalay CI method, and then to explore the synergistic protective effect for intestinal barrier and to explore the underlying mechanism.

In this study, we utilized the oxidative stress model in vitro and in vivo [31] to explore the synergistic antioxidative protective effects of SeMet and allicin focusing on the underlying mechanisms. Specifically, we investigated the relationship between antioxidant protection and the Nrf2 signaling pathway. Moreover, the interplay between the intestinal barrier, oxidative stress, and ER stress provides insight into the development of combination drugs based on SeMet and allicin.

## 2. Materials and Methods

### 2.1. Reagents

RPMI 1640 medium, fetal bovine serum (FBS), trypsin, and penicillin/streptomycin were purchased from Gibco, Life Technologies (Carlsbad, CA, USA). Diquat and fluorescein isothiocyanate (FITC)-dextran (FD4) were purchased from Sigma-Aldrich (Shanghai, China). Cell counting kit-8 (CCK-8) was obtained from Biosharp (Nanjing, China). The SOD, MDA, NO, GSSG, GSH and ROS reagent kits were acquired from Beijing Solarbio Science and Technology (Beijing, China). RNAiso Plus, PrimeScript™ RT reagent Kit with gDNA Eraser, and TB Green^®^ Premix Ex Taq™ II (Tli RNaseH Plus) were purchased from TaKaRa (Tokyo, Japan). Rabbit monoclonal antibody Nrf2 (ab92946) and mouse monoclonal antibody β-actin (ab8226) were purchased from Abcam (Cambridge, UK). Rabbit monoclonal antibody PERK (A21255), rabbit polyclonal antibody GRP78 (A0241), and rabbit polyclonal antibody CHOP (A0221) were purchased from Abclonal (Wuhan, China). Rabbit polyclonal antibody NQO1 (bs-2184R), rabbit polyclonal antibody Keap1(bs-3648R), Rabbit polyclonal antibody Occludin (bs-10011R), and Rabbit polyclonal antibody ZO-1 (bs-1329R) were purchased from Bioss (Beijing, China). HRP goat anti-rabbit IgG (AS014) was purchased from Abclonal (Wuhan, China), HRP goat anti-mouse IgG (bs-40296G-HRP) was purchased from Bioss (Beijing, China), and ML385, an Nrf2 inhibitor, was purchased from TargetMol (Boston, USA). SeMet (98%) was provided by Shanghai Yuanye Bio-Technology Co., Ltd (Shanghai, China). Allicin (alliin and alliinase powder mixture, purity 1.603%) was provided by Xinjiang Ailexin Pharmaceutical Co., Ltd (Xinjiang, China). 

### 2.2. Allicin Preparation

Alliinase catalyzes the conversion of alliin to allicin. Allicin stock solutions for in vitro and in vivo experiments were prepared from powder mixtures at concentrations of 160 μg/mL and 2 mg/mL, respectively, according to previous descriptions [32,33].

### 2.3. Cell Culture

Intestinal epithelial porcine cell line (IPEC-J2) was purchased from American type culture collection (ATCC) and preserved at the Nanjing Agricultural University. IPEC-J2 cells were cultured in RPMI 1640 medium (Gibco) supplemented with 10% FBS, 100 units/mL penicillin, and 100 μg/mL streptomycin in a 5% CO_2_ humidified atmosphere at 37 °C.

### 2.4. H_2_O_2_-Induced IPEC-J2 Cells: Oxidative Damage and SeMet + Allicin Treatment 

#### 2.4.1. Establishment of Oxidative Stress Model and Screening of Optimal Drug Combination 

IPEC-J2 cells were seeded into 96-well plates at 8000 cells/well. To build an oxidative stress injury model, the cells were treated with H_2_O_2_ (50, 100 and 200 μM) for 6 h. To determine the optimal SeMet and allicin concentration, IPEC-J2 cells were subjected to various concentrations of SeMet (0.01, 0.04, 0.16, 0.64, 2.56, 10.24, and 40.96 μg/mL) or allicin (0.5, 1, 2, 4, 6, and 8 μg/mL) for 24 h. To find the optimal combination under H_2_O_2_ treatment, IPEC-J2 cells were treated with different reagents: (1) Different doses of SeMet (0.01, 0.04, 0.16, and 0.64 μg/mL) or allicin (1, 2, 4, and 8 μg/mL) were pretreated for 12 h, then treated with (or without) 100 μM of H_2_O_2_ for 6 h; (2) Different doses of allicin (1, 2, 4 and 8 μg/mL) and a certain concentration of SeMet (0.04 μg/mL) were pretreated for 12 h, and then treated with (or without) 100 μM of H_2_O_2_ for 6 h, and each dose was repeated in five separate wells. Subsequently, the CCK-8 assay was conducted as previously [34].

#### 2.4.2. Determination of Combination Index (CI) Values

To investigate the interaction of SeMet and allicin combinations, the Chou-Talalay method was used to calculate the combination index (CI) values by describing the dose–effect curves of individual compound using CompuSyn software (version 1.0) [35]. The CI values of two compounds were evaluated using the formula—CI = (D)_1_/(DX)_1_ + (D)_2_/(DX)_2_—in which the (DX)_1_ and (DX)_2_ are the doses of sample 1 and sample 2 alone that inhibit x%. Also, the (D)_1_ and (D)_2_ show the portion of sample 1 and sample 2, in which their combination inhibition is x%. When CI = 1, the interaction between SeMet and allicin was an additive effect; when CI < 1, the interaction between SeMet and allicin reflected a synergistic effect, with the smaller CI showing the stronger synergistic effect; when CI > 1, the interaction between SeMet and allicin indicated an antagonistic effect [36].

#### 2.4.3. Cell Groups and Treatment

The groups were classified as follows: (1) Control; (2) H_2_O_2_; (3) SeMet; (4) Allicin; (5) SeMet + allicin. Cells were pretreated with SeMet (0.04 μg/mL) or allicin (2 μg/mL) for 12 h and treated with (or without) H_2_O_2_ (100 μM) for 6 h. In addition, the SeMet + allicin group was pretreated with an Nrf2 inhibitor ML385 (10 μM) alone for 12 h.

#### 2.4.4. Measurement of ROS in IPEC-J2 Cells

ROS generation level was measured using a fluorescence microscope and a ROS assay kit. Cells were treated with 10 μM of 2,7-dichlorodihydrofluorescein diacetate (DCFH-DA) for 20 min at 37 °C, and the cells were washed with a serum-free medium three times. Fluorescence intensity was detected in a ZEISS Axioscope 5 inverted fluorescence microscope (ZEISS, Oberkochen, Germany) at excitation and emission wavelengths of 488 nm and 525 nm, respectively. ROS generation level was quantified using flow cytometry [16]. Briefly, the ROS assay kit was used, and the same treatment was performed as described above, then, 10,000 cells were collected, and the fluorescence intensity of the cells was detected at 488 nm excitation wavelength and 525 nm emission wavelength using flow cytometer (BD LSRFortessa, Franklin Lakes, NJ, USA).

#### 2.4.5. Measurement of oxidative stress markers

IPEC-J2 cells were seeded into a 100 mm cell-culture dish at 10^6^ cells/dish. Cells were collected into the centrifuge tube, and an extracting solution (Tris hydrochloride, Tris-HCl) was added for ultrasonication on ice. The supernatants were collected on ice to test SOD activities, MDA, NO, GSH, and GSSG concentrations following the manufacturer’s instructions [37,38,39,40,41].

#### 2.4.6. Q-PCR 

The mRNA expression level was analyzed by quantitative real-time PCR (q-PCR) in IPEC-J2 cells. Total RNA was extracted from IPEC-J2 cells using an RNAiso Plus kit (TaKaRa, Osaka City, Japan), and the total RNA was reverse transcribed with PrimeScript™ RT reagent Kit with gDNA Eraser kit (TaKaRa, Japan) following the manufacturer’s instructions. The data were collected with the CFX96 Real-Time PCR Detection System (Bio-Rad, Hercules, CA, USA) and analyzed with Bio-Rad CFX, managed via the cycle threshold (2^−ΔΔCt^) method. Each sample reacted with five replicates. Table 1 lists the specific *Nrf2*, *NQO1*, *GRP78*, *IRE1*, *PERK*, *XBP1*, *ATF4*, *CHOP*, *ATF6*, and *β-actin* primers. 

#### 2.4.7. Western Blot

The protein expression levels of IPEC-J2 were detected by performing Western blot [42]. The cellular total protein and nuclear protein were extracted [43]. The proteins were adjusted to the same concentration after measuring the concentration by the BCA kit. The homogenous proteins were subjected to sodium dodecyl sulfate (SDS) electrophoresis. The proteins were separated and transferred to polyvinylidene difluoride (PVDF) membranes (Bio-Rad); the membranes were blocked with 5% skim milk in tris-buffered saline with tween (TBST) solution for 2 h at room temperature. Primary antibodies were incubated (1:1000) at 4 °C overnight while incubating the second antibodies (1:5000) at room temperature for 2 h. Immunoreactivity was visualized using chemiluminescence with an enhanced chemiluminescence (ECL) kit (Merck Millipore, Burlington, MA, USA).

#### 2.4.8. Wound Closure Assay

IPEC-J2 cells were seeded into 6-well plates at 2 × 10^5^ cells/well for 12 h. After treatment with allicin, SeMet, and ML385, the yellow pipette tips were used to make a vertical wound on the cell monolayer. The shedding cell fragments were gently washed away, and then cells were treated with SeMet and allicin for 12 h then treated with 100 μM of H_2_O_2_ for 6 h. The cell morphology was observed under a phase-contrast microscope with a 20 × objective (Leica DM IL LED, Germany). ImageJ software was used to analyze the data.

#### 2.4.9. Transepithelial Electrical Resistance (TEER) and Permeability Assay

IPEC-J2 cells were seeded in culture transwells (Corning; membrane area: 1.12 cm^2^; pore size: 0.4 μm) and placed into 12-well plates. When the TEER was stabilized (approximately 2000 Ω cm^2^), cells were pretreated as previously described in Section 2.4.3. The TEER was measured using the Millicell^®^ERS-2 (Merck Millipore, Darmstadt, Germany). After the last measurement of TEER value, 100 μL of FD4 (1 mg/mL) was added to the apical side of transwells (upper compartments). Cells were cultured for 30 min at 37 °C. Then, 100 μL of medium from the basolateral compartment (lower compartments) was collected to determine FD4 flux by detecting fluorescence intensity at an excitation wavelength of 484 nm and an emission wavelength of 535 nm, using a synergy HTX multifunctional microplate detector (BioTek Synergy HTX, Santa Clara, CA, USA) according to the standard curve (Figure S2). 

### 2.5. Diquat-Induced Intestinal Barrier Injury and SeMet + Allicin Treatment In Vivo 

#### 2.5.1. Establishment of Oxidative Stress Animal Model 

Male Balb/c mice aged 6 weeks and weighing 17–21 g were obtained from the Charles River laboratory. All mice experiments followed the Nanjing Agricultural University ethics committee guidelines (NJAU.NO20211103162). First, the diquat-induced oxidative stress model was established in our pre-experiment. The mice aged 6 weeks were habituated for one week in cages, with each cage containing five mice having free access to water and food. Fifteen mice were allocated to three groups: PBS, 12.5 mg/kg diquat, and 25 mg/kg diquat. After intraperitoneal injection of diquat for 24 h, samples (jejunum, liver, and blood) were collected from all mice. 

#### 2.5.2. Mice Groups and Treatment

**Figure 1:**
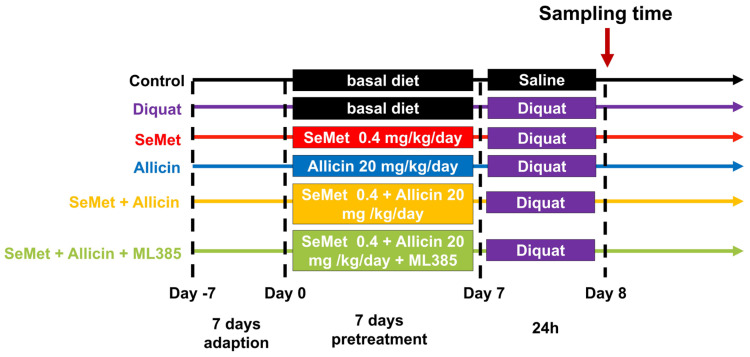
Scheme of animal experiment.

Mice were randomly divided into six groups (4 mice/group): control group, diquat group, SeMet + diquat group, allicin + diquat group, SeMet + allicin + diquat group, and SeMet + allicin+ ML385 + diquat group. Figure 1 shows that all mice were adapted for a week. The mice in the SeMet + diquat group, SeMet + allicin + diquat group and SeMet + allicin+ ML385 + diquat group received 0.4 mg/kg of SeMet by oral administration once a day for seven days. The mice in the allicin + diquat group, SeMet + allicin + diquat group, and SeMet + allicin+ ML385 + diquat group received 20 mg/kg of allicin by oral administration once a day for seven days. Meanwhile, mice in the SeMet + allicin+ ML385 + diquat group received a daily ML385 (30 mg/kg; dissolved in PBS with 5% DMSO) by intraperitoneal injection for seven days. The mice were treated with 25 mg/kg of diquat by intraperitoneal injection except the control group (treated with equivalent PBS). After another 24 h, blood was collected from the eyeballs of mice, collected into 1.5 mL EP tubes, and mice were euthanized by cervical dislocation to collect blood and jejunal samples. Finally, 4 h before sampling, four mice in each group were randomly selected and treated orally with 40 mg/mL of FD4 to assess intestinal barrier damage.

#### 2.5.3. Assessment of the Intestinal Permeability of FD4

The blood was kept in a refrigerator at 4 °C overnight and then centrifuged at 3000× *g* for 10 min using a microcentrifuge (Eppendorf 5425, Hamburg, Germany). The fluorescence of serum was detected using a synergy HTX multifunctional microplate detector at 485 and 535 nm excitation and emission wavelengths, respectively. Serum FD4 concentration was calculated using the standard curve (Figure S2). 

#### 2.5.4. SOD and MDA assay 

Jejunum samples of 0.1 g were weighed, and 1 mL of extracting solution was added for homogenization in an ice bath. Samples were centrifuged at 8000× *g* at 4 °C for 10 min, and then the supernatant was collected. The SOD activity and MDA concentration were directly detected as per protocol.

#### 2.5.5. Histopathological Examination

All jejunum samples were fixed in a 4% paraformaldehyde solution, embedded in paraffin, cut into slides, and stained with hematoxylin and eosin (H&E). Slides were scanned with a scanner (3DHISTECH PANNORAMIC MIDI II, Budapest, Hungary) and analyzed with ImageJ (version 1.48).

#### 2.5.6. ROS Determination in Jejunum 

Fresh mouse jejunum segments were quickly frozen at −80 °C, and jejunum samples were sliced and stained with dihydroethidium (DHE) at 37 °C for 30 min. Subsequently, all sections were washed with PBS three times. After that, the cell nuclei were stained with 4′,6-diamidino-2-phenylindole (DAPI) for 15 min and washed with PBS three times. Finally, the sections were sealed and scanned by the scanner above.

#### 2.5.7. Western Blot Analysis of Related Pathway Proteins in Jejunum Tissue

The jejunum tissue total proteins were extracted, and the protein expression levels of jejunum tissue were detected by Western blot assay as previously described in Section 2.4.7.

### 2.6. Statistical Analysis

Data analysis was conducted using GraphPad Prism 8.0.2 software, reporting data as mean ± SD. Statistical significance was analyzed using one-way analysis of variance (ANOVA); *p* < 0.05 was considered statistically significant.

## 3. Results

### 3.1. Candidate H_2_O_2_, SeMet or Allicin Dosage under Selection

**Figure 2:**
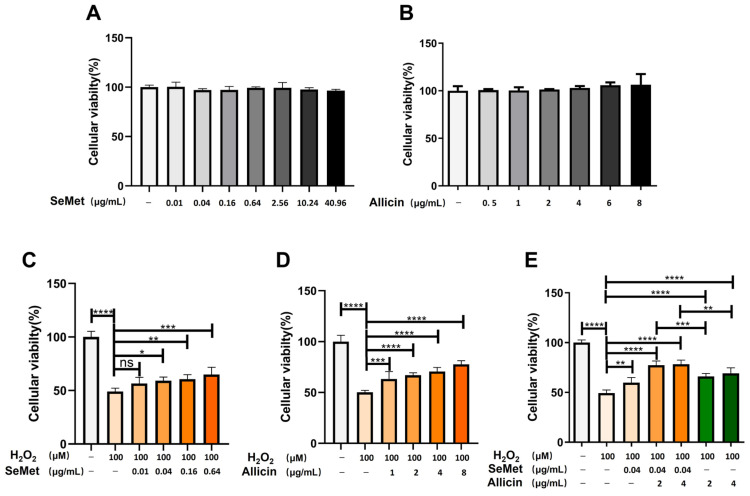
Effects of SeMet, allicin, or both on IPEC-J2 cell viability. (**A**,**B**) The effect of SeMet or allicin on the viability of IPEC-J2 cells after 12 h treatment. (**C**–**E**) Cell viability of IPEC-J2 cells in SeMet, allicin, and SeMet + allicin treatment for 12 h, followed by 100 μM of H_2_O_2_ or PBS (control group) for 6 h. Values are expressed as mean ± S.D. The symbols for the significance of differences: * *p* < 0.05; ** *p* < 0.01; *** *p* < 0.001; **** *p* < 0.0001; ns, insignificant. The same symbols represent the figures below.

Appropriate oxidative stress leads to epithelial cell damage. Consistent with findings from previous studies [44], our previous experiments have proven that the 100 μM H_2_O_2_ decreased cell viability, increased ROS production, induced cell apoptosis, decreased the SOD activity, and increased MDA content (Figure S1A–G). This oxidative stress model in vitro was applied to subsequent experiments. Consistent with previous findings, different concentrations of SeMet (0.01–40.96 μg/mL) or allicin (0.5–8 μg/mL) were added to IPEC-J2 cell proliferation to select the optimal dosage [45,46]. After treatment with SeMet or allicin for 12 h, cell viability was measured (Figure 2A,B), revealing an insignificant reduction compared with the control. 

### 3.2. SeMet, Allicin, or Both Alleviates H_2_O_2_-Induced IPEC-J2 Cell Damage

The potential protection of SeMet and allicin on IPEC-J2 cells was investigated using the CCK-8 method. IPEC-J2 cells were exposed to different concentrations of SeMet (0.01–0.64 μg/mL) or allicin (1–8 μg/mL) for 12 h before being treated with 100 μM of H_2_O_2_ for 6 h. Figure 2C,D illustrate different cell viability, respectively. After being treated with H_2_O_2_, cell viability decreased. However, the pretreatment of SeMet (0.04, 0.64, and 0.64 μg/mL) or allicin (1, 2, 4, and 8 μg/mL) significantly increased cell viability. Surprisingly, SeMet (0.04 μg/mL) and allicin (2 and 4 μg/mL) co-treatment significantly increased the cell survival rate compared with SeMet or allicin treatment separately (Figure 2E). The results suggest that SeMet and allicin could synergistically attenuate cytotoxicity caused by H_2_O_2_. 

### 3.3. Synergistic Effect of SeMet and Allicin Combination

To confirm that SeMet and allicin synergistically attenuate cell damage through oxidative stress, the combination index (CI), introduced by Chou and Talalay, was used to quantitatively evaluate drug combination effects as antagonism effects (CI > 1), additive effects (CI = 1), or synergism effects (CI < 1). The CI values of the SeMet (0.04 μg/mL) and allicin (2 and 4 μg/mL) combination are less than 1. The results suggest that SeMet and allicin has a synergistic protective effect on IPEC-J2 cells at this concentration, as well as the optimal drug combination was SeMet (0.04 μg/mL) and allicin (2 μg/mL) (Table 2).

### 3.4. SeMet, Allicin, or Both Regulated the Generation of ROS and Redox Balance

**Figure 3:**
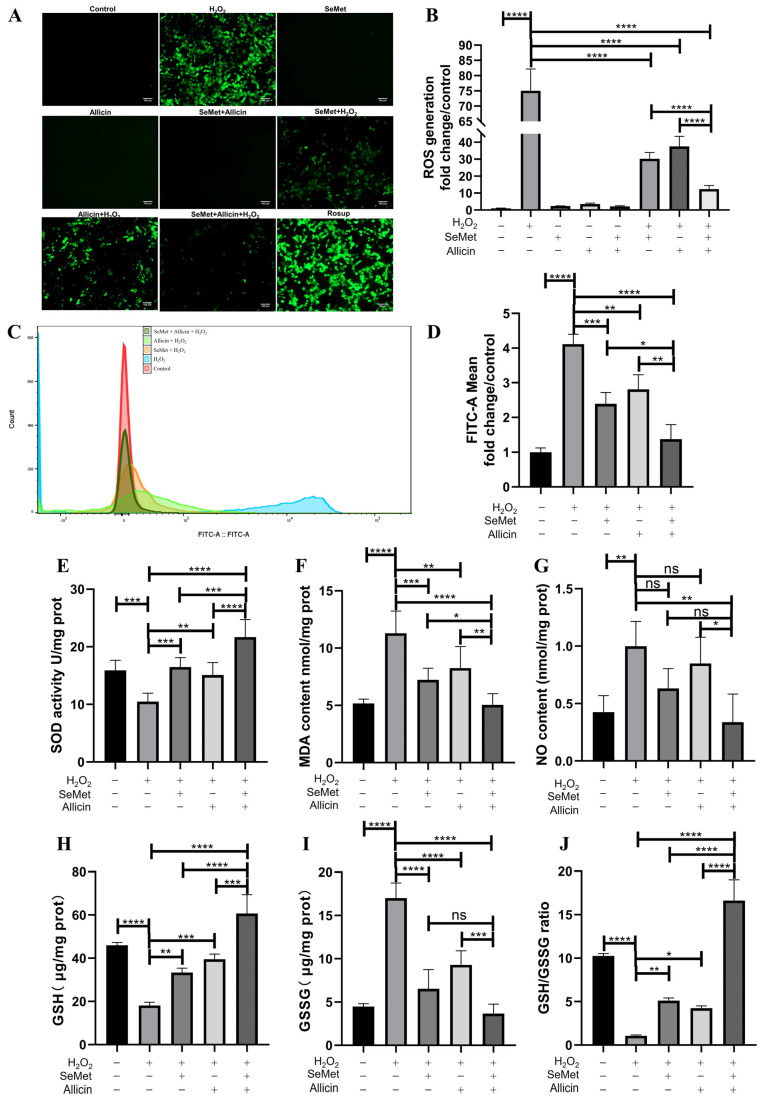
Effect of SeMet, allicin, or both on ROS generation and redox homeostasis in IPEC-J2 cells induced by H_2_O_2_. (**A**,**B**) IPEC-J2 cells were pretreated with SeMet, allicin, or both for 12 h and 100 μM of H_2_O_2_ for 6 h. Intracellular ROS generation is visualized using fluorescence microscope and fluorescence intensity analysis. “Rosup” is short for ROS positive inducer. (**C**,**D**) Flow cytometry data and the quantification of fluorescence intensity are shown. IPEC-J2 cells were lysed by ultrasonication and centrifuged, and then the supernatants were collected to detect the (**E**) SOD activity, (**F**) MDA content, (**G**) NO content, (**H**) GSH content, (**I**) GSSG content, and (**J**) GSH/GSSG ratio. The protein concentration was regulated to be equal. Values are expressed as mean ± S.D. The symbols for the significance of differences: * *p* < 0.05; ** *p* < 0.01; *** *p* < 0.001; **** *p* < 0.0001; ns, insignificant.

Next, we investigated the effect of inhibiting SeMet, allicin, or both on ROS generation in IPEC-J2 cells. Fluorescence images of ROS stained by DCFH-DA implied that H_2_O_2_ exposure significantly increased intracellular ROS generation compared with the control group. SeMet and allicin play an significant lightening role in producing intracellular ROS (Figure 3A,B). This aligns with the flow cytometry analysis: SeMet and allicin, either alone or in combination, exhibit cytoprotective effects against H_2_O_2_ induced cellular stress by significantly attenuating intracellular ROS production (Figure 3C,D). The SeMet + allicin + H_2_O_2_ group significantly decreased ROS levels compared with the SeMet + H_2_O_2_ or allicin + H_2_O_2_ group. ROS production is the marker of oxidative stress, and some antioxidant enzymes and intermediate products indicate the redox reaction. The results indicated that H_2_O_2_ treatment significantly decreased SOD activity, GSH content, and GSH/GSSG ratio compared with the control group, which was reversed by SeMet and allicin, alone or in combination. Moreover, the SeMet + allicin + H_2_O_2_ group significantly increased the SOD activity, GSH content, and GSH/GSSG ratio compared with the H_2_O_2_ group and SeMet + H_2_O_2_ or allicin+ H_2_O_2_ groups (Figure 3E,H,J). Meanwhile, H_2_O_2_ treatment significantly increased MDA, NO, and GSSG content compared with the control group. SeMet and allicin, alone or in combination, significantly decreased MDA and GSSG content. Furthermore, the SeMet + allicin + H_2_O_2_ group significantly decreased MDA, NO, and GSSG content compared with the H_2_O_2_ group and the allicin + H_2_O_2_ group (Figure 3F,G,I). These results elucidate the synergistic effect of SeMet and allicin on antioxidative stress parameters.

### 3.5. SeMet, Allicin, or Both Activate the Nrf2 Signaling Pathway 

**Figure 4:**
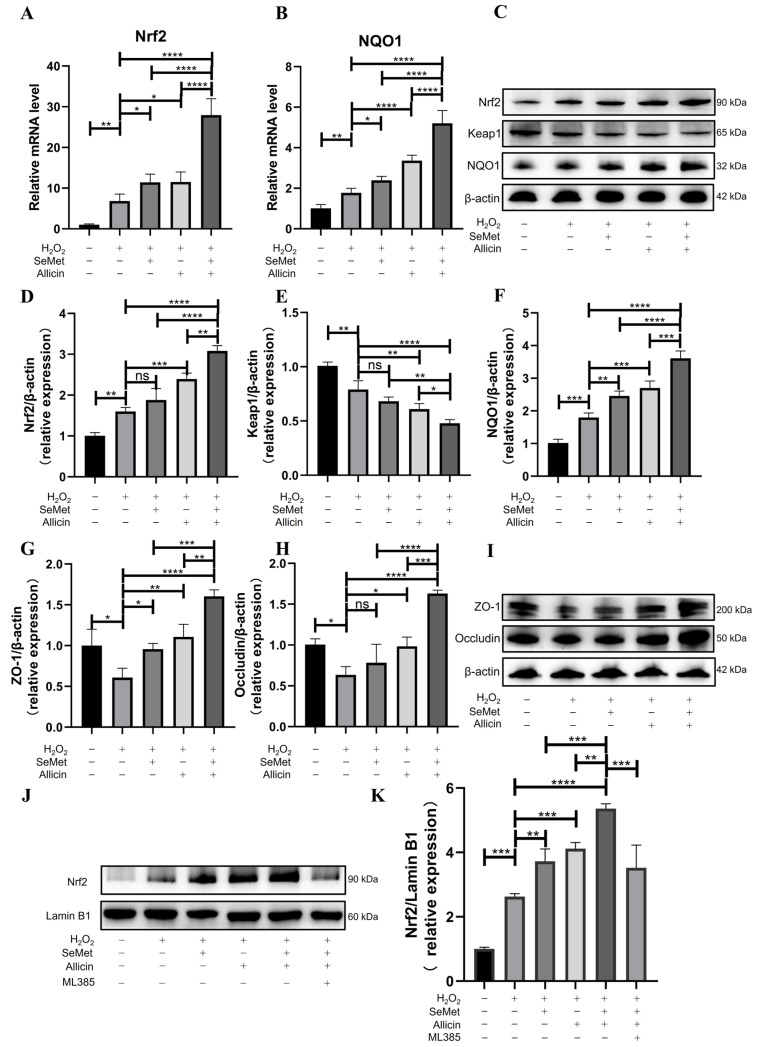
Effects of SeMet, allicin, or both in Nrf2 antioxidant pathway, Nrf2 nuclear translocation, and tight junction protein in IPEC-J2 cells. (**A**,**B**) IPEC-J2 cells were pretreated with SeMet, allicin, or both for 12 h and 100 μM of H_2_O_2_ for 6 h. The Nrf2 relative gene expression (Nrf2 and NQO1) was monitored by the q-PCR analysis. (**C**–**I**) Nrf2, NQO1, Keap1, ZO-1, and Occludin expressiona in total protein of cells were detected and analyzed by Western blot. (**J**,**K**) Cell nuclear proteins were separated, and Western blot analysis was conducted to determine the Nrf2 expression in the nucleus. Values are expressed as mean ± S.D. The symbols for the significance of differences: * *p* < 0.05; ** *p* < 0.01; *** *p* < 0.001; **** *p* < 0.0001; ns, insignificant.

Q-PCR showed that the *Nrf2* and *NQO1* mRNA expression was significantly up-regulated in cells after being stimulated with H_2_O_2_ for 6 h compared with the control group, (Figure 4A,B). SeMet and allicin, alone or in combination, promoted the *Nrf2* and *NQO1* mRNA expression. The SeMet + allicin + H_2_O_2_ group significantly improved *Nrf2* and *NQO1* mRNA expression levels compared with H_2_O_2_, allicin + H_2_O_2,_ or SeMet + H_2_O_2_ group. Similarly, the Western blot showed that SeMet or allicin could enhance the protein expression of Nrf2 and NQO1 and decrease that of Keap1 (Figure 4C–F). SeMet + allicin + H_2_O_2_ group significantly improved Nrf2 and NQO1 protein expression compared with the H_2_O_2_, SeMet + H_2_O_2_, or allicin + H_2_O_2_ groups (Figure 4D,F). Conversely, the SeMet + allicin + H_2_O_2_ group significantly decreased Keap1 protein expression compared with the H_2_O_2_, allicin + H_2_O_2_, or SeMet + H_2_O_2_ groups (Figure 4E). Nrf2 separated from Keap1 and translocated to the nucleus, and then combined with the ARE to protect cells from oxidative damage. The subsequent results suggest that SeMet and allicin synergistically promoted the efficiency of the nuclear translocation of Nrf2 (Figure 4J,K).

### 3.6. SeMet, Allicin, or Both Increase TJ Protein Expression

Furthermore, we investigated TJ protein expression levels in IPEC cells. Western blot indicated that SeMet or allicin significantly enhanced ZO-1 and Occludin protein expression. However, H_2_O_2_ exposure significantly decreased their expression. The SeMet + allicin + H_2_O_2_ group significantly increased ZO-1 and Occludin protein expression compared with the H_2_O_2_, SeMet + H_2_O_2_, or allicin + H_2_O_2_ groups (Figure 4G–I). These results suggested that SeMet and allicin served a synergistic function to strengthen TJ protein integrity.

### 3.7. SeMet, Allicin, or Both Attenuate ERS

**Figure 5:**
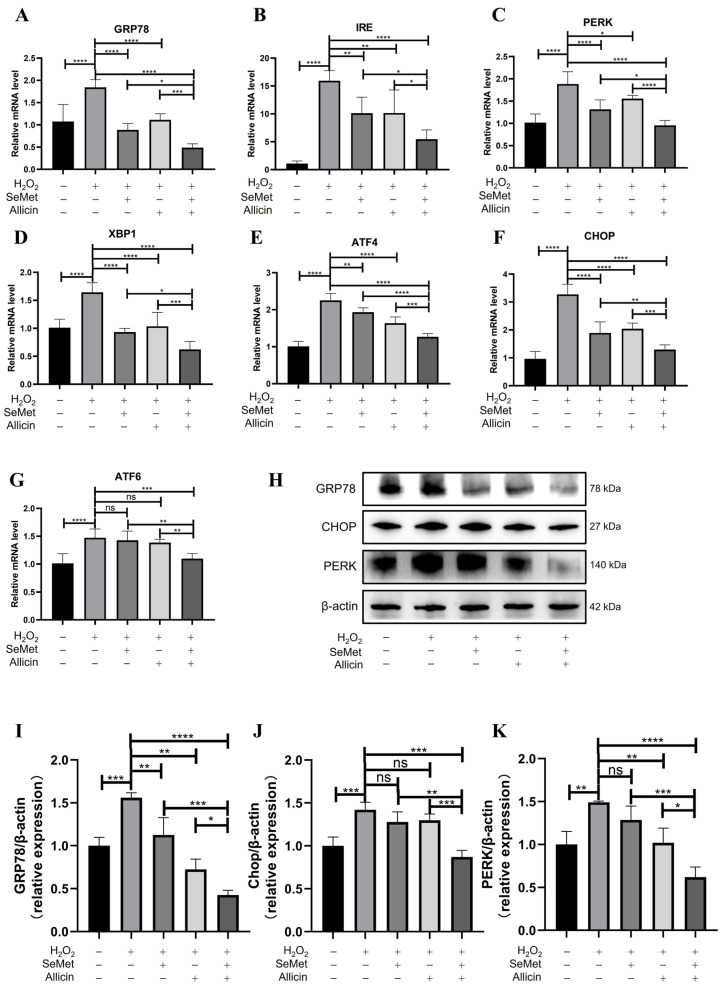
Effects of SeMet or/and allicin on ER stress. (**A**–**G**) GRP78, IRE, PERK, XBP1, ATF4, CHOP, and ATF6 expression were detected by q-PCR. (**H**–**K**) GRP78, PERK, and CHOP expression were detected and analyzed by Western blot. Values are expressed as mean ± S.D. The symbols for the significance of differences: * *p* < 0.05; ** *p* < 0.01; *** *p* < 0.001; **** *p* < 0.0001; ns, insignificant.

Figure 5A–G show that, compared with the control group_,_ the mRNA expression of molecular chaperone *GRP78* and ERS-related genes—*IRE1*, *PERK*, *XBP1u*, *ATF4*, *CHOP*, and *ATF6*—were significantly up-regulated in the H_2_O_2_ group. However, SeMet or allicin significantly down-regulated *GRP78*, *IRE1*, *PERK*, *XBP1u*, *ATF4*, and *CHOP* mRNA expression. Moreover, the SeMet + allicin + H_2_O_2_ group significantly down-regulated the mRNA expression levels of the above ERS-related genes compared with the H_2_O_2_, SeMet + H_2_O_2_, or allicin + H_2_O_2_ group. Similarly, Western blot showed that SeMet or allicin could significantly reduce GRP78 protein expression. However, H_2_O_2_ exposure significantly increased their GRP78, PERK, and CHOP expression. The SeMet + allicin + H_2_O_2_ group could significantly decrease GRP78, PERK, and CHOP protein expression compared with the H_2_O_2_, SeMet + H_2_O_2,_ or allicin + H_2_O_2_ group (Figure 5H–K). The results implied that SeMet and allicin synergistically alleviated the expression of the ERS-related protein.

### 3.8. SeMet and Allicin Synergistically Activate Nrf2 Signaling Pathway

**Figure 6:**
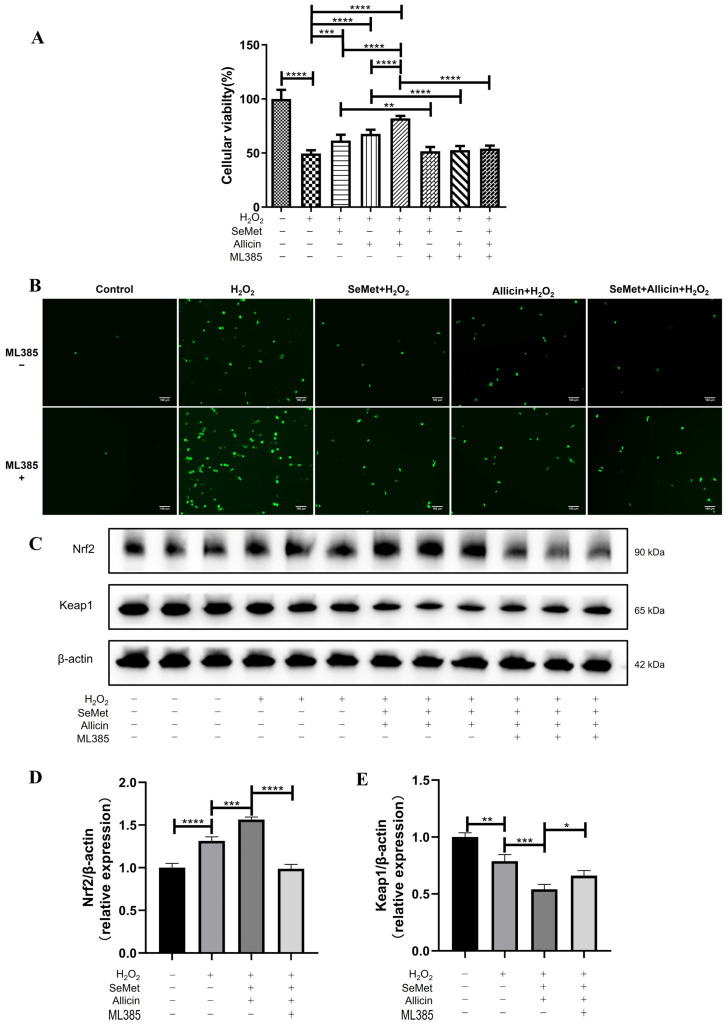
Effects of nuclear translocation of Nrf2 on SeMet and allicin attenuating oxidative stress. IPEC-J2 cells were pretreated or untreated with ML385 (an Nrf2 inhibitor) for 12 h. Meanwhile, cells were treated with SeMet, allicin, or both for 12 h and 100 μM of H_2_O_2_ for 6 h. (**A**) Cell viability. (**B**) ROS level. (**C**–**E**) The relative expression levels of Nrf2 and keap1 after H_2_O_2_ treatment. Values are expressed as mean ± S.D. The symbols for the significance of differences: * *p* < 0.05; ** *p* < 0.01; *** *p* < 0.001; **** *p* < 0.0001; ns, insignificant.

To further explore the importance of the Nrf2 signaling pathway, IPEC-J2 cells were exposed to ML385 (an Nrf2 inhibitor) for 12 h and incubated with SeMet and allicin together. When IPEC-J2 cells were treated with ML385, the cells were more vulnerable to H_2_O_2_ than without ML385, even though they were treated with SeMet or allicin (Figure 6A). Moreover, ML385 attenuated the protective effect of SeMet and allicin in preventing ROS generation by managing levels of H_2_O_2_ (Figure 6B). Furthermore, ML385 abrogated the increasing Nrf2 protein expression and decreasing Keap1 protein expression with SeMet and allicin treatment (Figure 6C–E). These results confirmed that the synergetic effect on antioxidation of SeMet and allicin was achieved by activating the Nrf2 pathway.

### 3.9. SeMet and Allicin Improve Cell Migration and Maintain Barrier Function Depending on Nrf2 Activation

**Figure 7:**
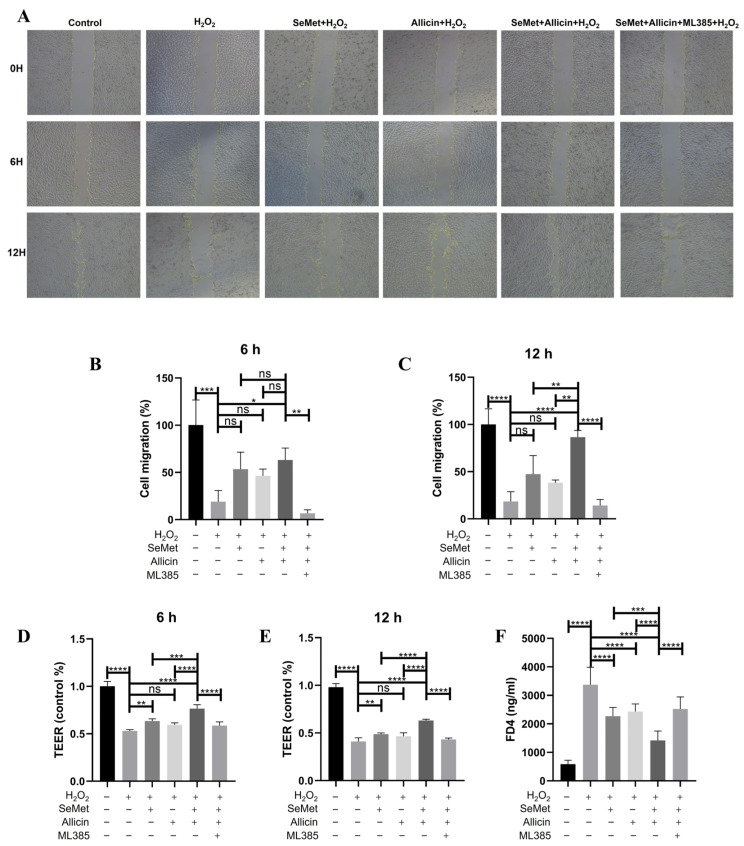
Effects of Nrf2 on IPEC-J2 cell migration and permeability. IPEC-J2 cells. (**A**–**C**) IPEC-J2 cells were pretreated with SeMet, allicin, and ML385 for 12 h and 100 μM of H_2_O_2_ for 6 or 12 h. IPEC-J2 cell migration ability was detected in each group by scratch assay (50×), and the scratched place was analyzed. (**D**,**E**) When the TEER was stabilized (approaching 2000 Ω cm^2^), the IPEC-J2 cells were pretreated with SeMet, allicin, and ML385 for 12 h before treatment with 100 μM of H_2_O_2_ for 6 or 12 h. The TEER was detected at 6 or 12 h. (**F**) Simultaneously, the FD4 permeability of IPEC-J2 cells was measured at 12 h. Values are expressed as mean ± S.D. The symbols for the significance of differences: * *p* < 0.05; ** *p* < 0.01; *** *p* < 0.001; **** *p* < 0.0001; ns, insignificant.

Cell migration is vital for the damage repair and barrier integrity of intestinal epithelial cells. H_2_O_2_ exposure reduced IPEC-J2 cell migration capacity, whether treated for 6 or 12 h; however, SeMet or allicin promoted migration. Moreover, the SeMet + allicin + H_2_O_2_ group significantly increased cell migration compared with the SeMet + H_2_O_2_ or allicin + H_2_O_2_ group (Figure 7A–C). However, the synergistic promotion of cell migration of SeMet and allicin was interdicted by ML385. When IPEC-J2 cells grew for 20 days, the intestinal barrier was simulated. To evaluate the protective role of SeMet, allicin, or both on barrier permeability, TEER permeation was measured after H_2_O_2_ exposure for 6 or 12 h, and FD4 was measured after H_2_O_2_ exposure for 12 h. The results showed that H_2_O_2_ exposure significantly reduced TEER at 6 or 12 h, while it was relieved by SeMet or allicin administration. Furthermore, the SeMet + allicin + H_2_O_2_ group significantly increased TEER permeation compared with SeMet + H_2_O_2_ or allicin + H_2_O_2_ group (Figure 7D,E). Meanwhile, FD4 permeability was increased by H_2_O_2_ exposure at 12 h. However, SeMet, allicin, or both reversed the increase in FD4 content. Moreover, the SeMet + allicin + H_2_O_2_ group significantly decreased FD4 content compared with SeMet + H_2_O_2_ or allicin + H_2_O_2_ group (Figure 7F). However, the synergistic effect was abolished by ML385. Based on these results, we corroborated that SeMet and allicin synergistically enhanced the tissue repair ability and the protective effect for the barrier. However, this synergistic protective effect depended on Nrf2-mediated antioxidative function.

### 3.10. SeMet and Allicin Improve Tight Junction and Inhibit ER Stress Depends on Nrf2 Activation 

**Figure 8:**
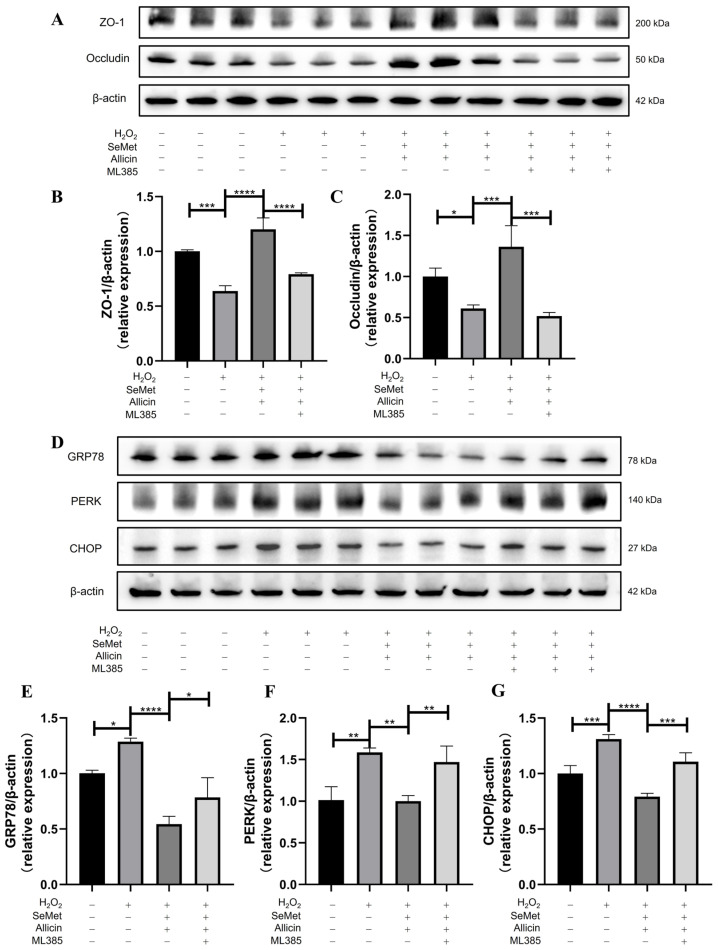
Effects of nuclear translocation of Nrf2 on the tight junction protein and ER stress in IPEC-J2 cells. IPEC-J2 cells were pretreated with SeMet, allicin, and ML385 for 12 h and H_2_O_2_ for 6 h. (**A**–**C**) Western blot was used to detect and analyze the expression of ZO-1, Occludin, (**D**–**G**) GRP78, PERK, and CHOP. Values are expressed as mean ± S.D. The symbols for the significance of differences: * *p* < 0.05; ** *p* < 0.01; *** *p* < 0.001; **** *p* < 0.0001; ns, insignificant.

The Western blot indicated that SeMet and allicin could significantly enhance ZO-1 and Occludin expression, but the reverse effect of protein expression was blocked by ML385 (Figure 8A–C). Similarly, Western blot suggested that SeMet and allicin could significantly ameliorate GRP78, PERK, and CHOP protein expression, but ML385 reversed the decrease in these protein expressions (Figure 8D–G). These above results elucidated that SeMet and allicin inhibited ER stress and improved the tight junction by synergistically activating the Nrf2 signal pathway.

### 3.11. SeMet, Allicin, or Both Increased the Intestinal Function and Alleviated Intestinal Injury in Mice

**Figure 9:**
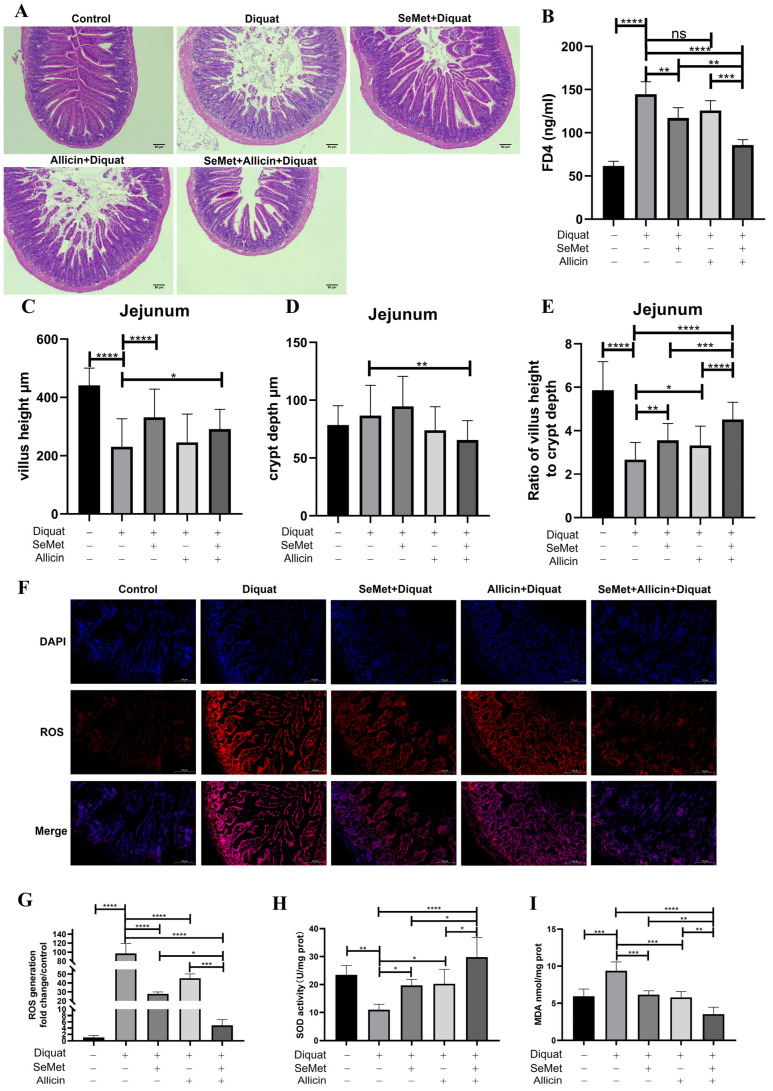
Effect of SeMet and allicin on the jejunum barrier function and antioxidative capacity. (**A**,**C**–**E**) Villous height, crypt depth, and relative ratios of villous height to crypt depth in the jejunal tissues. (**B**) Effect of SeMet and allicin on the serum FD4 content. (**F**,**G**) ROS production was quantified and analyzed. (**H**,**I**) The jejunum samples were homogenized and centrifuged, and then the supernatants were collected to detect SOD activity and MDA content (*n* = 4). Values are expressed as mean ± S.D. The symbols for the significance of differences: * *p* < 0.05; ** *p* < 0.01; *** *p* < 0.001; **** *p* < 0.0001; ns, insignificant.

To prove whether SeMet or allicin alleviate intestinal barrier injury in vivo, diquat was used as an oxidative stress inducing drug [16]. Our previous experiments proved that 25 mg/kg body weight of diquat caused more sensitive oxidative stress in the jejunum than in the blood and liver via intraperitoneal injection (Figures S3A–F and S4A,B). Similar to the results of previous studies, 0.4 mg/kg of SeMet and 20 mg/kg of allicin was orally administered in our study [16,33]. In this study, Figure 9A illustrates that the SeMet + diquat group and allicin + diquat group increased villous height and decreased crypt depth compared with the diquat group in the morphological observation. The diquat group decreased the villous height and decreased the ratio of villous height to crypt depth compared to the control group. However, SeMet + allicin + diquat group significantly increased the ratio of villous height to crypt depth compared with the diquat group, SeMet + diquat group, and allicin + diquat group (Figure 9C–E). Figure 9B showed that the diquat group increases serum FD4 content compared with the control group, showing that oxidative stress generated severe intestinal barrier injury. Pretreatment with allicin alone did not effectively inhibit the diquat-induced FD4 increase. However, SeMet can significantly reduce FD4 production in blood under the challenge of diquat. Nevertheless, the FD4 content in the SeMet + allicin + diquat group was significantly lower than in the diquat group, SeMet + diquat group, and allicin + diquat group. These results demonstrate that SeMet acts in concert with allicin to increase intestinal barrier function in vivo.

### 3.12. SeMet, Allicin, or Both Improve Antioxidative Capacity in Jejunum of Mice

Dihydroethidium (DHE) was used to stain the mouse jejunum for detecting ROS production. Diquat significantly stimulated ROS generation, which was significantly abolished by SeMet and allicin pretreatment. Moreover, the ROS generation in the SeMet + allicin + diquat group was significantly lower than in the SeMet + diquat group and allicin + diquat group (Figure 9F,G). The results indicated that diquat treatment progressively decreased SOD activity in the control group. SeMet and allicin, either alone or in combination, significantly increased SOD activity. SeMet + allicin + H_2_O_2_ group significantly increased SOD activity compared with the diquat group, SeMet + diquat group, and allicin + diquat group. Meanwhile, diquat treatment significantly increased MDA content compared with the control group. SeMet and allicin, alone or in combination, significantly decreased MDA content. A significant decrease in MDA in the SeMet + allicin + H_2_O_2_ group was observed compared with the diquat group, SeMet + diquat group, and allicin + diquat group (Figure 9H,I). These results show that the SeMet and allicin synergistically increase antioxidant capacity in vivo.

### 3.13. SeMet and Allicin Synergistically Activate the Nrf2 Pathway to Improve Barrier Function and Inhibit ER Stress in the Jejunum of Mice

**Figure 10:**
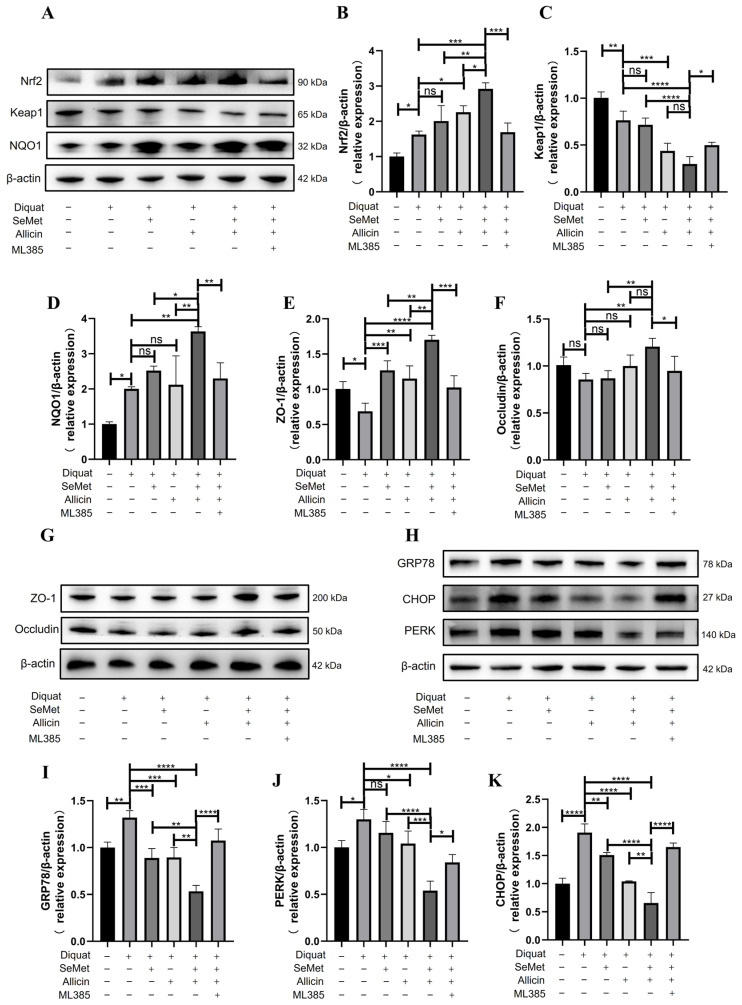
SeMet and allicin synergistically activate the Nrf2 pathway to mitigate diquat-induced oxidative stress and ER stress in mice. (**A**–**D**) Western blot was used to detect and analyze the expression of the Nrf2 signaling pathway (Nrf2, NQO1, and Keap1), (**H**–**K**) ER stress pathway (GRP78, PERK, and CHOP), and (**E**–**G**) tight junction protein (ZO-1 and Occludin) (*n* = 4). Values are expressed as mean ± S.D. The symbols for the significance of differences: * *p* < 0.05; ** *p* < 0.01; *** *p* < 0.001; **** *p* < 0.0001; ns, insignificant.

The above in vitro experiments have demonstrated that SeMet and allicin synergistically promoted Nrf2 nuclear translocation and activated downstream antioxidant signaling pathways, thereby protecting the intestinal barrier and attenuating ER stress. We performed Western blot to confirm the mechanism in vivo (Figure 10A,G,H and Figure S5A–E). The results indicated that Nrf2 and NQO1 expression increased and Keap1 expression decreased in the jejunum of mice in the diquat group. However, SeMet and allicin, either alone or in combination, further activated Nrf2 and NQO1 expression and decreased Keap1 expression. However, when ML385 was added, the effect of SeMet and allicin was significantly restricted or even rendered ineffective (Figure 10A–D). Additionally, TJ protein (ZO-1) expression was decreased in the diquat group compared with the control group, while SeMet and allicin, alone or in combination, ameliorated the damage to the ZO-1 protein. Similarly, ML385 blocked the positive effect of SeMet and allicin for TJ protein (Figure 10E,F). Subsequently, GRP78, PERK, and CHOP expression increased in the diquat group. Nevertheless, SeMet and allicin, either alone or in combination, decreased GRP78, PERK, and CHOP expression; ML385 reversed the decline of these protein expressions (Figure 10H–K). These results show that SeMet and allicin synergistically activated the Nrf2 pathway to maintain barrier integrity and inhibit ER stress in vivo.

## 4. Discussion

Oxidative stress ubiquitously occurs in the gut of humans and animals. The intestine is susceptible to excessive ROS. The intestinal epithelium, a significant natural barrier, protects the body against hazardous or detrimental substances under normal physiological circumstances by preserving the integrity of epithelial morphology and functions [47]. Therefore, the inhibition of oxidative injury is key to improving epithelial function. Being a safe and efficient Se source, SeMet could be nonspecifically incorporated into proteins to replace Met, consequently being an endogenous Se pool for selenoprotein synthesis. Allicin is the main active ingredient in garlic; it has anti-inflammation, anti-infection, and antioxidative stress effects. An interesting study shows that the selenylation modification of garlic polysaccharide is a novel method with an immune-enhancing activity [48,49]. However, whether this modification can increase the antioxidant effect of garlic polysaccharides remains unclear. Allicin has a stronger ability than garlic polysaccharides in terms of antioxidant function. Whether allicin and SeMet can serve a synergistic antioxidant function in vitro and in vivo is still unknown, although the antioxidant protection of SeMet or allicin alone was confirmed [50,51,52]. 

H_2_O_2_ and diquat are employed as the stress inducers in this experiment due to their stability in vitro and in vivo. Here, the optimum SeMet (0.04 μg/mL) and allicin (2 μg/mL) concentrations were first screened, and the results showed that SeMet and allicin, either alone or combined, can mitigate oxidative damage. Furthermore, SeMet positively correlates with allicin in elevating antioxidant protection. ROS elevation causes molecular damage and cell death [26]. Meanwhile, SeMet and allicin can synergistically mitigate ROS generation and enhance SOD activity level, GSH content, and GSH/GSSG ratio while decreasing MDA and GSSG content. These results suggest that the co-treatment of SeMet and allicin synergistically enhances cellular antioxidant capacity compared to their individual treatments, similar to the results of many previous studies [48,49,53,54,55]. Likewise, SeMet and allicin can synergistically improve oxidative stress of jejunum in mice.

**Figure 11:**
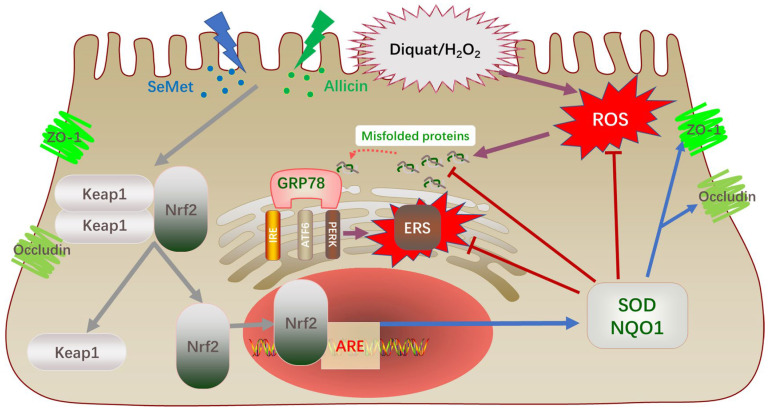
The schematic diagram of SeMet and allicin synergistically activate the Nrf2 pathway and attenuate ER stress. SeMet and allicin were transported into the cells and dissociated Keap1 and Nrf2. Nrf2 translocated into the nucleus to form an Nrf2-Maf heterodimer binding to ARE and activated downstream antioxidant enzymes expression (NQO1 and SOD). This mechanism can inhibit the production of ROS and strengthen TJ protein integrity. The synergistic effect of SeMet and allicin increased the cellular antioxidant level and reduced the UPR response caused by oxidative stress. Then, GRP78 rebound with the three signal proteins (PERK, IRE, and ATF6) to inhibit the occurrence of ER stress.

The Nrf2-ARE signaling pathway is an important cellular defense mechanism against oxidative stress injury. Previous studies have clarified that the interaction of GSH and Nrf2 increased keratinocyte survival and accelerated wound healing [56]. Chang et al. illustrated that allicin can attenuate the inflammation of diabetic macroangiopathy by up-regulating Nrf2 [57]. Li et al. elucidated Se yeast-protected kidneys from mycotoxin-induced oxidative damage via motivating Nrf2/Keap1 pathways in chickens [58]. In this study, q-PCR and Western blot results showed that after being exposed to H_2_O_2_ or diquat, mRNA and protein expression levels of Nrf2 and NQO1 were up-regulated. It could be an auto-regulatory mechanism for the antioxidant capacity of cells. However, this negative feedback regulation was insufficient to protect cells from oxidative stress at supraphysiological concentrations. Therefore, self-adaptation does not provide adequate antioxidant protection. Succeeding SeMet and allicin significantly enhanced the mRNA and protein expression levels of Nrf2 and NQO1 but decreased Keap1 protein expression, suggesting that the Nrf2 pathway was significantly activated. Additionally, we detected the nucleus translocation of Nrf2 to dig deeper into the details of the activated process of Nrf2 pathway. We separated the nuclear proteins and analyzed the relative transcript level of Nrf2. The data illustrated that SeMet and allicin synergistically enhanced the nuclear translocation of Nrf2 to exert a cytoprotective mechanism (Figure 11) [16,59,60]. To further illustrate the key role of the Nrf2 pathway in anti-oxidative effects in IPEC-J2 cells, an Nrf2 inhibitor, ML385, was used in vitro and in vivo [61]. Consistent with Singh et al. and Liu et al., ML385 down-regulated Nrf2 and NQO1 protein expression compared with untreated cells, evidenced by Western blot, and then weakened Nrf2 transcriptional activity [62]. Additionally, cellular antioxidant protection was blocked when ML385 abolished Nrf2. Therefore, the Nrf2 pathway was critical to keep intracellular redox equilibrium to counteract excessive oxidative stress [63]. 

The rate of damage repair and function of the intestinal barrier is related to its redox status [4]. Herein, H_2_O_2_ exposure reduced IPEC-J2 cell migration capacity and TEER while increasing FD4 permeability and down-regulating ZO-1 and Occludin protein expression; SeMet and allicin reversed these effects. When ML385 was used to abrogate the Nrf2 signal, the synergistic protective effect of SeMet and allicin on the intestinal barrier was lost. Likewise, SeMet and allicin increased the intestinal barrier functions and alleviated intestinal injury in vivo, which was reduced by ML385, aligned with the cell experiment above. In current study, H_2_O_2_ exposure up-regulated *GRP78*, *IRE1*, *PERK*, *XBP1*, *ATF4*, *CHOP*, and *ATF6* mRNA expression levels in IPEC-J2 cells, which were down-regulated by SeMet and allicin. The results above show that oxidative stress can indeed cause ER stress; however, the synergistic effect of SeMet and allicin in attenuating ER stress is still unclear. We hypothesized that the combined effect of SeMet and allicin activated the Nrf2 pathway, increased the level of cellular antioxidants, decreased ROS production, and reduced the UPR response caused by H_2_O_2_ exposure. Once the misfolded protein becomes correctly folded, it will not competitively bind GRP78 with the three signal proteins (PERK, IRE, and ATF6), thereby inhibiting ER stress (Figure 11). Likewise, SeMet and allicin alleviated intestinal ER stress in vivo, which was abolished by ML385, as shown in the WB results. Therefore, the above results show that SeMet and allicin can synergically increase antioxidant capacity, improve intestinal barrier function and alleviate ERS through the activation of the Nrf2 antioxidant pathway.

In conclusion, SeMet and allicin can synergistically alleviate oxidant stress injury, enhance intestinal barrier function and the activity of antioxidant enzymes, and attenuate ER stress by activating the Nrf2 signaling pathway. Although we have found that SeMet and allicin can synergically improve the efficiency of Nrf2 translocation to the nucleus, the exact molecular mechanism is unknown. More drug combinations with synergistic antioxidant effects remain to be discovered. These findings provide a theoretical basis for further clinical combination of SeMet and allicin to overcome oxidative-stress-related intestinal injury.

## Figures and Tables

**Table 1:** *Nrf2*, *NQO1*, *GRP78*, *IRE1*, *PERK*, *XBP1*, *ATF4*, *CHOP*, *ATF6*, and β-actin primers.

Gene	Gene Accession Number	Primer Sequence 5′-3′
*Nrf2*	XM_021075133.1	F: CACCACCTCAGGGTAATA
		R: GACAAACATTCAAGCCGC
*NQO1*	XM_047791619.1	F: CCAGCAGCCCGGCCAATCTG
		R: AGGTCCGACACGGCGACCTC
*GRP78*	X92446.1	F: AATGGCCGTGTGGAGATCA
		R: GAGCTGGTTCTTGGCTGCAT
*IRE1*	XM_047757844.1	F: CTGCACTCCCTCAACATCGT
		R: GTAGGTGGGGTTCTCCTTGC
*PERK*	XM_003124925.4	F: ACTACAAGCGGGAAAGGAGC
		R: CACCAGTGCAAAAGGAGCAC
*ATF6*	XM_021089516.1	F: GCTCCTCCGTTCCTCCTTACCTC
		R: CTGACAACATGGGCTGCCTCTG
*XBP1*	XM_012934292.1	F: TGAAGAGGAGGCAGAGACCAAGG
		R: GGGAGATGTTCTGGAGGGGTGAC
*ATF4*	XM_021090887.1	F: GCCCCCGCAGATAGTGAA
		R: TGGGGAAAGGGGAAGAGTTTG
*CHOP*	XM_008978498.4	F: TCTGGCTTGGCTGACTGAGGAG
		R: CCGTTTCCTGGGTCTTCTTTGGTC
*β-actin*	XM_003124280	F: GATGAGATTGGCATGGCTTT
		R: CACCTTCACCGTTCCAGTTT

**Table 2:** The CI values of SeMet and allicin on the cell survival rate.

Compound	Dose SeMet (μg/mL)	Dose Allicin (μg/mL)	CI
SeMet + allicin	0.04	1	0.672 ± 0.495
		2	0.263 ± 0.232
		4	0.402 ± 0.307
		8	0.584 ± 0.560

CI values of each compound are presented at different proportion. The results are represented as means ± SD (*n* = 4).

## Data Availability

Data are contained within the article.

## Supplementary Materials

The following supporting information can be downloaded at: https://www.mdpi.com/article/10.3390/toxics12100719/s1, Figure S1: Effects of H_2_O_2_ on IPEC-J2 cell viability, apoptosis and redox homeostasis; Figure S2: The standard curve of FD4; Figure S3: Diquat induces serum, liver, and jejunum oxidative stress; Figure S4: Effects of diquat on redox homeostasis and morphology of jejunum; Figure S5: Additional images of Western Blot.

## Abbreviations

Se: Selenium; SeMet, Selenomethionine; Nrf2, Nuclear factor erythroid 2-related factor 2; KEAP1, Kelch-like ECH-associated protein 1; NQO1, NAD(P)H dehydrogenase [quinone] 1; SOD, Superoxide Dismutase; MDA, Malondialdehyde; GSH, Glutathione; GSSG, Oxidized glutathione; NO, Nitric oxide; ROS, Reactive oxygen species; ERS, Endoplasmic reticulum stress; GRP78, Glucose-regulated protein; IRE, Inositol-requiring enzyme; PERK, Protein kinase R-like ER kinase; XBP1u, X box-binding protein 1; ATF4, Activating transcription factor-4; CHOP, C/EBP-homologous protein; ATF6, Activating transcription factor 6; ARE, Antioxidant response element; ZO-1, Zona occludens protein 1; TJs, Tight junctions; UPR, Unfolded protein response; SD, Sprague Dawley; TRPA1, Transient receptor potential A1; FBS, Fetal bovine serum; FITC, Fluorescein isothiocyanate; CCK-8, Cell counting kit-8; IPEC-J2, Intestinal epithelial porcine cell line; DCFH-DA, 2,7-dichlorodihydrofluorescein diacetate; q-PCR, quantitative real-time PCR; SDS, Sodium dodecyl sulfate; PVDF, Polyvinylidene difluoride; TBST, Tris-buffered saline with tween; TEER, Transepithelial electrical resistance.
