# Supplementary material for: Selenomethionine and Allicin Synergistically Mitigate Intestinal Oxidative Injury by Activating the Nrf2 Pathway
Source: Toxics. 2024 Sep 30;12(10):719. doi: 10.3390/toxics12100719 (PMC11510923; doi:10.3390/toxics12100719)
Supplement: Supplementary file 1 [file toxics-12-00719-s001.zip › toxics-3162280-supplementary.pdf]

Supplementary figures

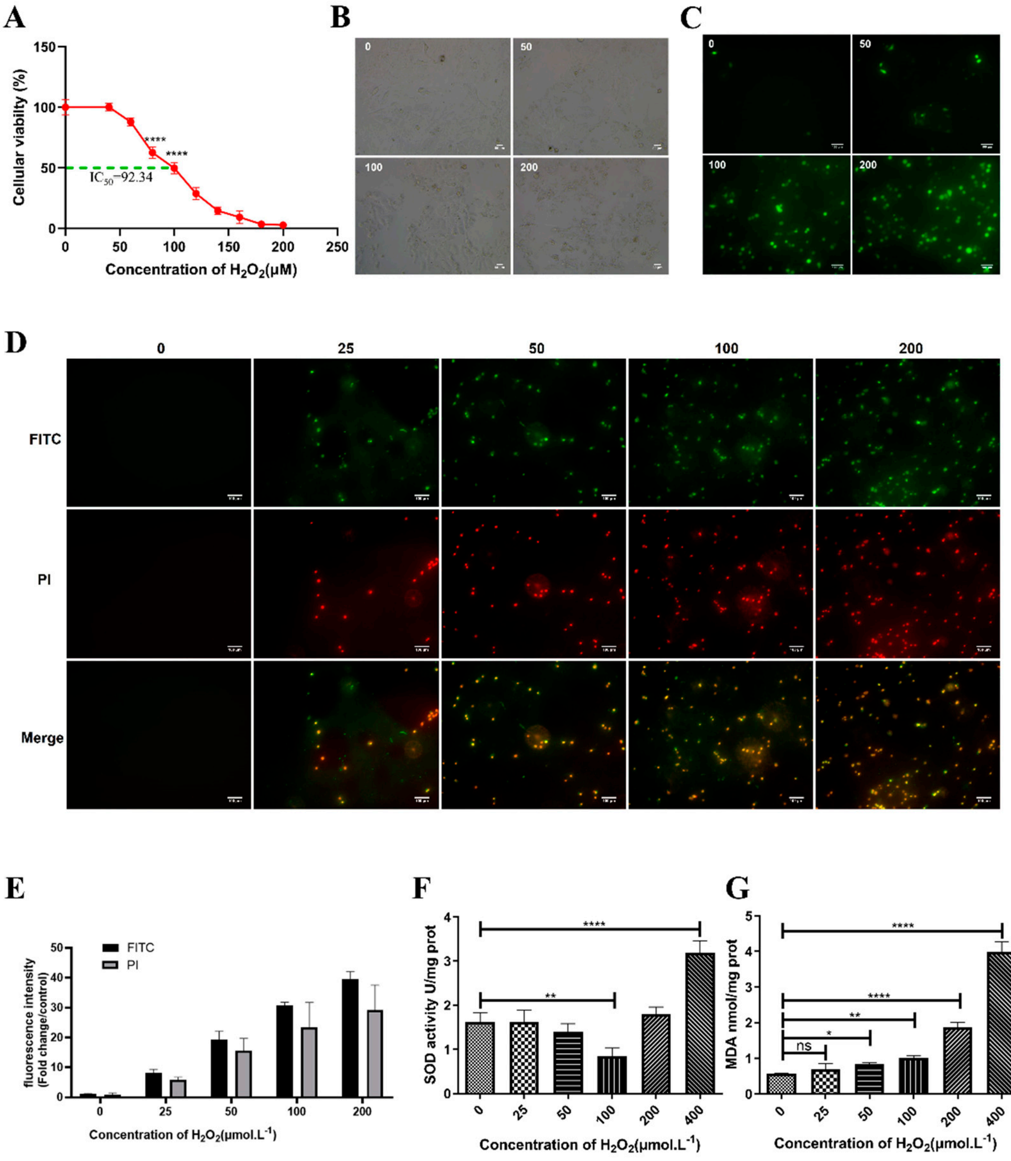

Figure S1. Effects of H_2O_2 on IPEC-J2 cell viability, apoptosis and redox homeostasis. (A) Effects of H_2O_2 on IPEC-J2 cell viability. The IPEC-J2 cells were treated with indicated H_2O_2 concentrations (0, 40, 60, 80, 100, 120, 140, 160, 180, and 200 µM) for 6 h. **(B)** Morphological changes of IPEC-J2 cells under an inverted phase contrast microscope; scale bar: 50 µm. **(C)** ROS was stained and visualized with an inverted fluorescence microscope. **(D)** Fluorescence images of AnnexinV FITC-stained and PI-stained apoptotic cells; scale bar:100 µm. **(E)** The average fluorescence intensity in the jejunum was quantified. **(F–G)** Redox indicator (SOD activity and MDA content).

A

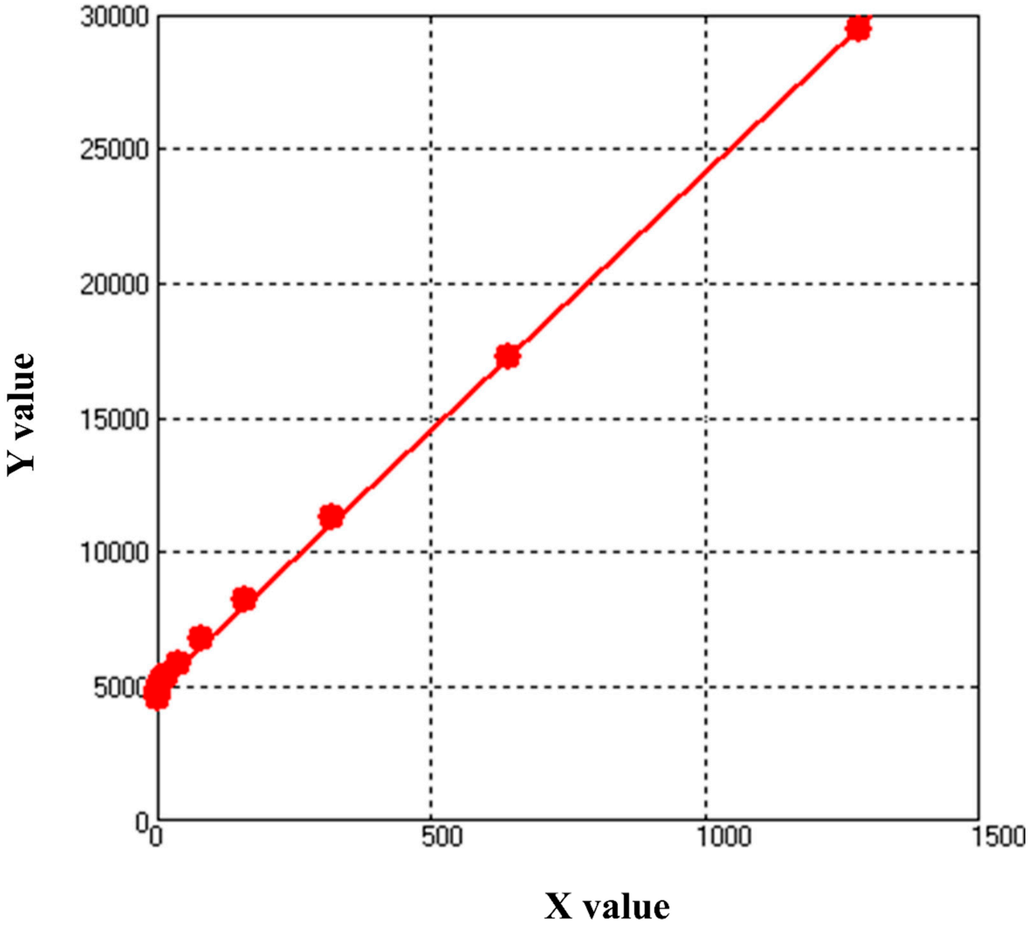

B

Equation of linear regression: y = a + b*x			
----------------------------------			
a =	4981.86421		
b =	19.19907		
r^2 =	0.99936		

Figure S2. The standard curve of FD4. (A–B) The standard curve (FD4 at 1.25, 2.5, 5, 10, 20, 40, 80, 160, 320, 640, and 1280 ng/mL concentrations) was established to calculate the FD4 concentration.

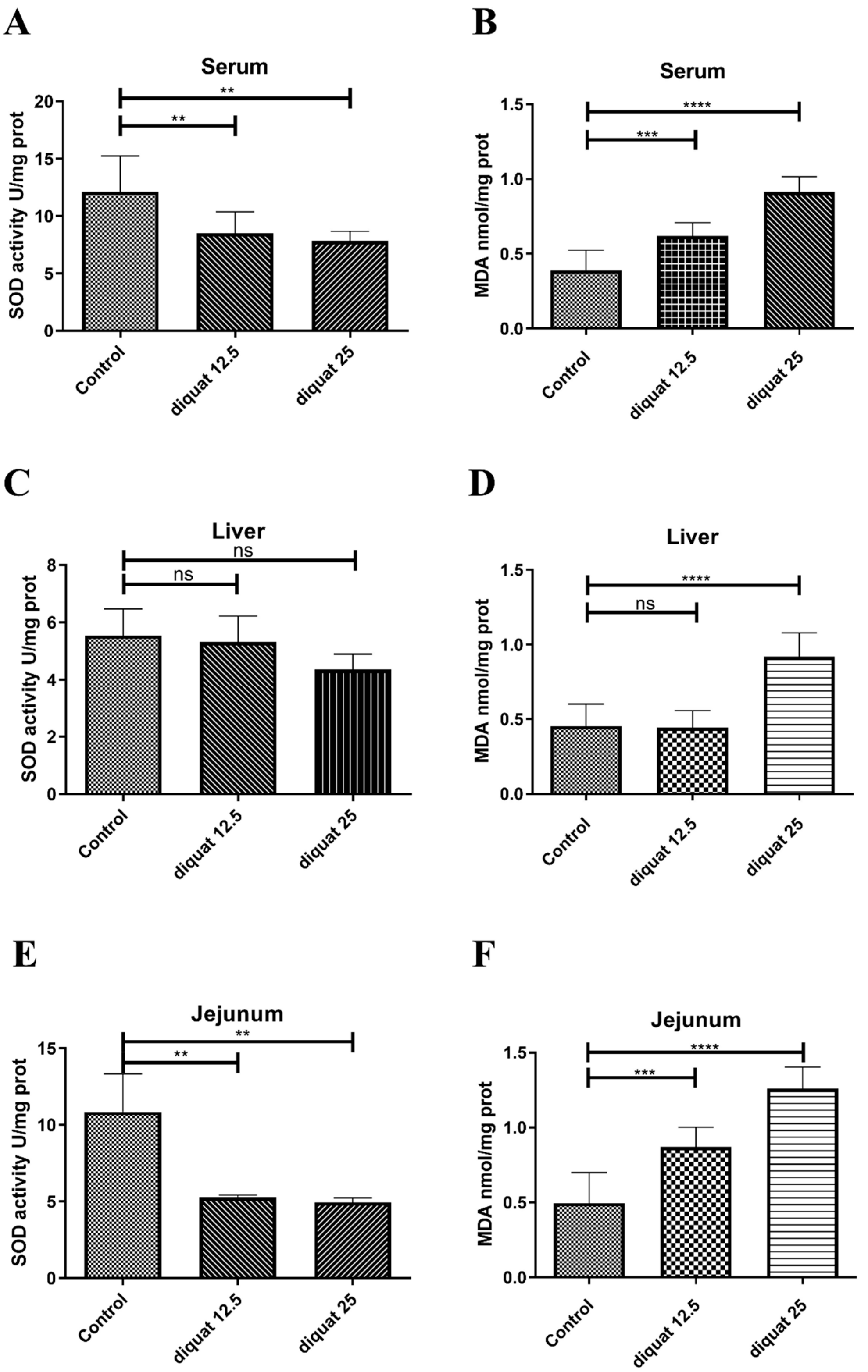

Figure S3. Diquat induces serum, liver, and jejunum oxidative stress. The liver or jejunum samples were homogenized and centrifuged, then the supernatants were collected to detect the **(C, E)** SOD activity and **(D, F)** MDA content. **(A)** The serum SOD activity and **(B)** MDA content were detected directly.

A

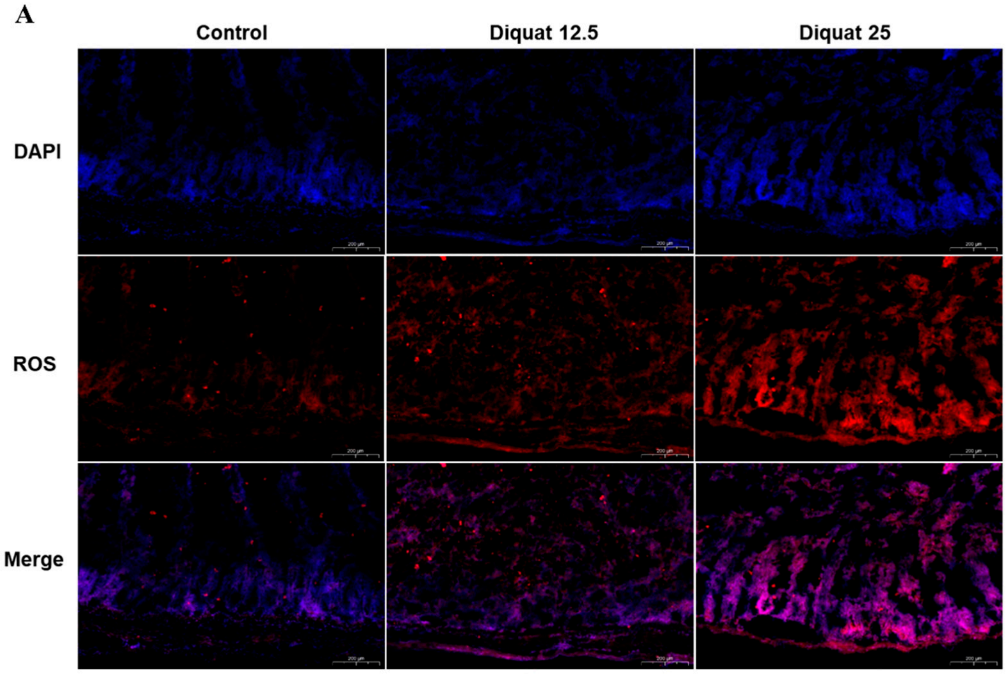

B

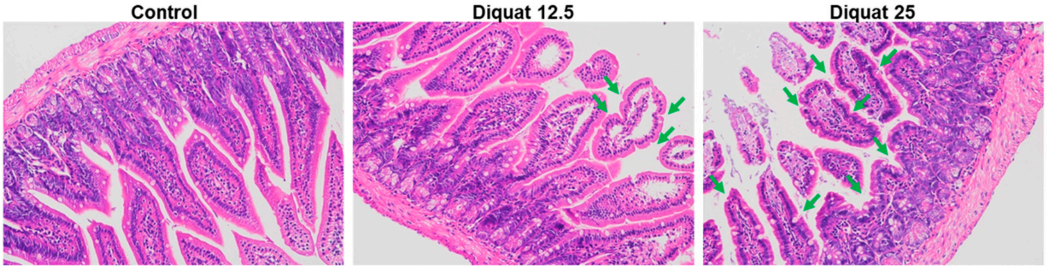

Figure S4. Effects of diquat on redox homeostasis and morphology of jejunum. (A) Fluorescence of ROS stained by DHE in jejunum. **(B)** Jejunum morphology.

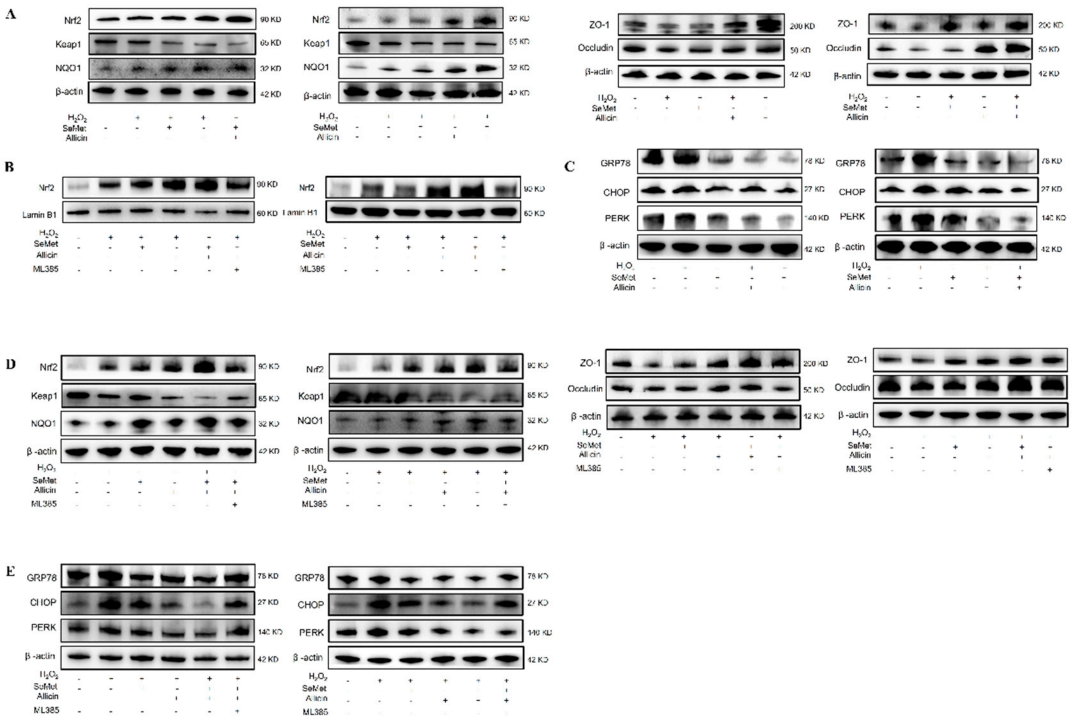

Figure S5. Additional images of Western Blot. IPEC-J2 cells were pretreated with SeMet and allicin for 12 h and H_2O_2 for 6 h. **(A)** Western blot detected and analyzed Nrf2, NQO1, Keap1, ZO-1, and Occludin expression. **(B)** Western blot analysis of the Nrf2 expression in the nucleus. **(C)** Western blot detected and analyzed GRP78, PERK, and CHOP expression. Mice were given SeMet and allicin orally and received a daily ML385 by intraperitoneal injection. The protein expression level of jejunum tissue was detected by western blot assay. Western blot detected and analyzed **(D)** the expression of the Nrf2 signaling pathway (Nrf2, NQO1, and Keap1), tight junction protein (ZO-1 and Occludin), and **(E)** ERS pathway (GRP78, PERK, and CHOP).
